# Short stature and hypoparathyroidism in a child with Kenny-Caffey syndrome type 2 due to a novel mutation in *FAM111A* gene

**DOI:** 10.1186/s13633-016-0041-7

**Published:** 2017-01-25

**Authors:** Mary B. Abraham, Dong Li, Dave Tang, Susan M. O’Connell, Fiona McKenzie, Ee Mun Lim, Hakon Hakonarson, Michael A. Levine, Catherine S. Choong

**Affiliations:** 1 Department of Endocrinology, Princess Margaret Hospital, Perth, Australia; 2 School of Paediatrics and Child Health, The University of Western Australia, Perth, Australia; 3 Center for Applied Genomics, Abramson Research Center, The Children’s Hospital of Philadelphia, Philadelphia, USA; 4 Telethon Kids Institute, Perth, Australia; 5 Genetic Services of Western Australia, Princess Margaret Hospital and King Edward Memorial Hospital, Perth, Australia; 6 School of Pathology and Laboratory Medicine, The University of Western Australia, Perth, Australia; 7 Department of Biochemistry, PathWest Laboratory Medicine, Perth, Australia; 8 Sir Charles Gairdner Hospital, Nedlands, Perth, Australia; 9 Division of Human Genetics and Department of Pediatrics, The Children’s Hospital of Philadelphia and The Perelman School of Medicine, Philadelphia, USA; 10 Division of Pulmonary Medicine, The Children’s Hospital of Philadelphia, Philadelphia, USA; 11 Division of Endocrinology and Diabetes, The Children’s Hospital of Philadelphia, Philadelphia, USA; 12 Center for Bone Health, The Children’s Hospital of Philadelphia, Philadelphia, USA; 13 Department of Endocrinology and Diabetes, Princess Margaret Hospital, Perth, Australia

**Keywords:** Kenny-Caffey syndrome Type 2, *FAM111A* gene, Growth hormone, Hypoparathyroidism, Short stature, Genetics

## Abstract

**Background:**

Hypoparathyroidism in children is a heterogeneous group with diverse genetic etiologies. To aid clinicians in the investigation and management of children with hypoparathyroidism, we describe the phenotype of a 6-year-old child with hypoparathyroidism and short stature diagnosed with Kenny-Caffey syndrome (KCS) Type 2 and the subsequent response to growth hormone (GH) treatment.

**Case presentation:**

The proband presented in the neonatal period with hypocalcemic seizures secondary to hypoparathyroidism. Her phenotype included small hands and feet, hypoplastic and dystrophic nails, hypoplastic mid-face and macrocrania. Postnatal growth was delayed but neurodevelopment was normal. A skeletal survey at 2 years of age was suggestive of KCS Type 2 and genetic testing revealed a novel *de novo* heterozygous mutation c.1622C > A (p.Ser541Tyr) in *FAM111A*. At 3 years and 2 months, her height was 80cms (SDS −3.86). She had normal overnight GH levels. GH therapy was commenced at a dose of 4.9 mg/m^2^/week for her short stature and low height velocity of 5cms/year. At the end of the first and second years of GH treatment, height velocity was 6.5cms/year and 7.2cms/year, respectively with maximal dose of 7.24 mg/m^2^/week.

**Conclusion:**

This case highlights the phenotype and the limited response to GH in a child with genetically proven KCS type 2. Long-term registries monitoring growth outcomes following GH therapy in patients with rare genetic conditions may help guide clinical decisions regarding the use and doses of GH in these conditions.

## Background

Hypoparathyroidism is an important cause of neonatal hypocalcemia presenting in the first week of life [1]. It is characterized by low or normal parathyroid hormone (PTH) levels in the presence of hypocalcemia and hyperphosphatemia. Although commonly seen as an iatrogenic complication following anterior neck surgery in adults, the etiology of hypoparathyroidism in children is more diverse and includes a heterogeneous group of disorders, many of which have a genetic basis [2, 3]. An increased understanding of the genetic etiology and improved genetic testing has provided an opportunity to expand the molecular diagnosis of hypoparathyroidism [2].

Kenny-Caffey syndrome (KCS) is an uncommon cause of hypoparathyroidism with one out of 37 patients reported with this condition in a cohort of children with primary hypoparathyroidism [3]. It is characterized by proportionate short stature along with cortical thickening and medullary stenosis of tubular bones, delayed closure of anterior fontanelle, eye abnormalities, and hypoparathyroidism. The autosomal dominant form or KCS Type 2 (OMIM 127000) caused by mutations in *FAM111A* [4] is distinguished from the autosomal recessive form (KCS Type 1), caused by mutations in tubulin-folding cofactor E (*TBCE*) gene, by the absence of microcephaly and mental retardation [5]. Awareness of the different phenotypes and the underlying genetic mutation improves the ability of the physician to undertake appropriate investigations, predict patient outcomes and provide appropriate genetic counselling [3]. We present the clinical characteristics of a 6-year-old girl with neonatal hypocalcemia, post-natal short stature, macrocrania and normal intellect diagnosed with KCS Type 2.

## Case presentation

**Fig. 1:**
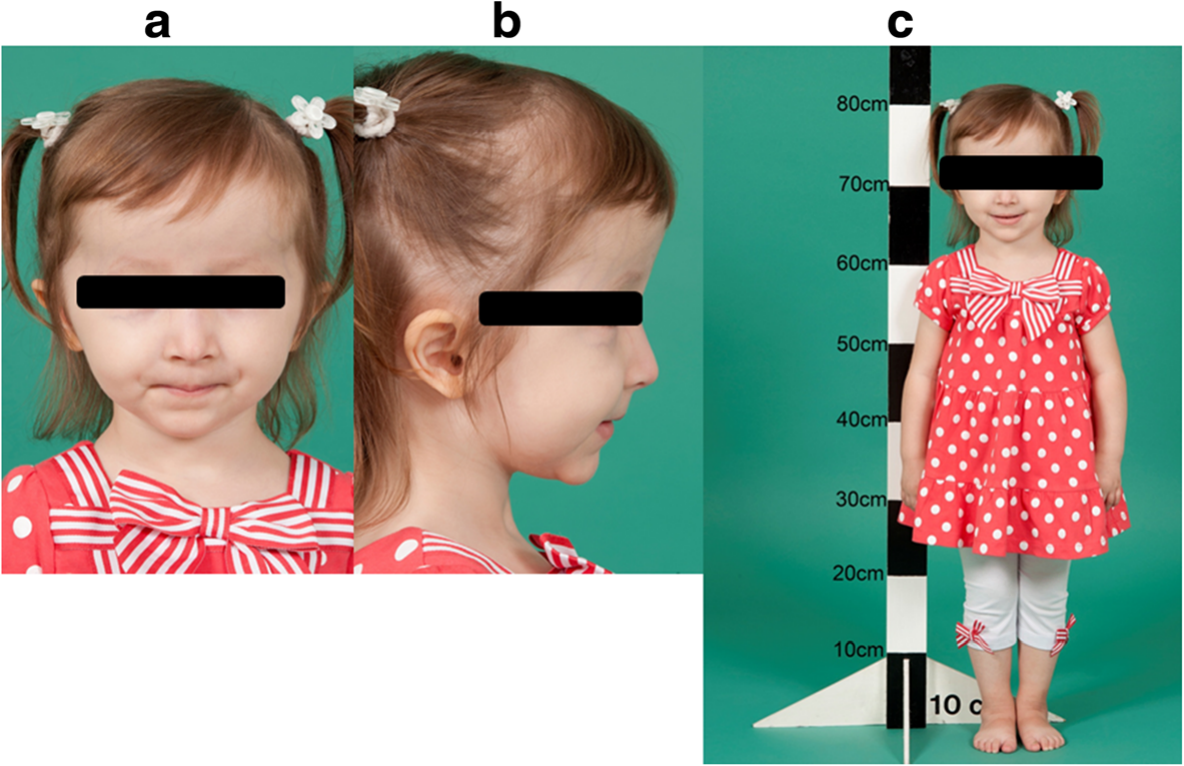
Figures 1**a** and 1**b** demonstrate the facial phenotype of the patient. Large head with persistent fontanels, telecanthus, small palpebral fissures, small pinched upturned nose with elfin facies. Figure 1**c** demonstrates the significant growth restriction at 3 years and 2 months of age

**Fig. 2:**
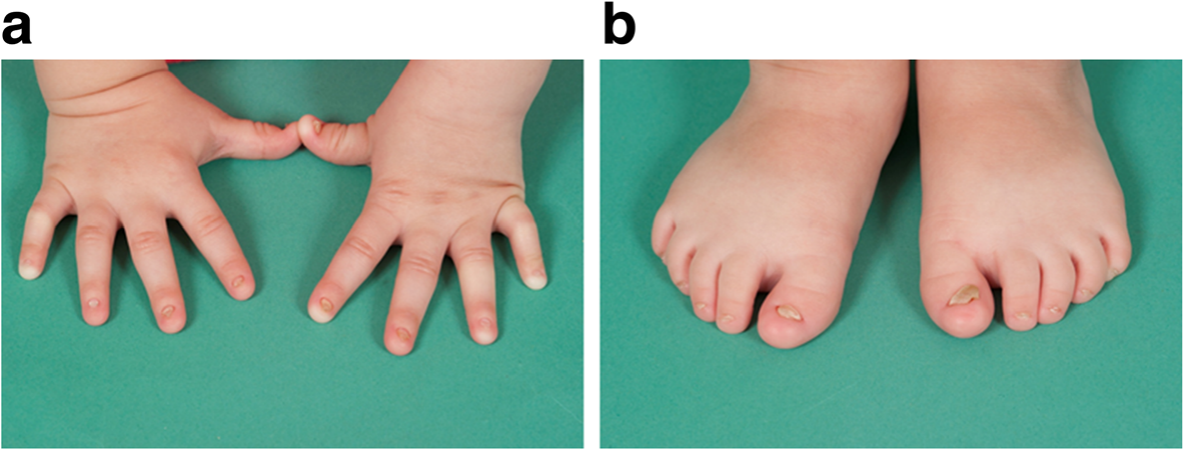
Figures 2**a** and 2**b** demonstrate the small hands and feet respectively with triangular dysplastic nails

The proband was born by natural conception to non-consanguineous Italian parents at 39 weeks of gestation by an elective caesarean section for breech delivery with a birth weight of 3.26kgs (SDS 0.1), length of 49cms (SDS −0.1) and head circumference of 36cms (SDS 1.8). She presented on day 8 of life with hypocalcemic seizures secondary to hypoparathyroidism (Table 1) with normal blood glucose and renal function. Normocalcemia was achieved with parenteral calcium gluconate infusion. Thereafter, supplementation with oral elemental calcium and calcitriol maintained appropriate serum levels of calcium and phosphate. Her phenotype included macrocrania and large persistent fontanelles. She had relatively elfin facies with hypoplasia of the mid-face, telecanthus, small palpebral fissures, a small pinched upturned nose and a small chin (Fig. 1a and b). Proportionate limb shortening with additional circumferential skin folds was present. Hands and feet were small with bilateral single palmar creases and triangular, hypoplastic and dystrophic nails (Fig. 2). An ophthalmological examination in the neonatal period did not reveal any abnormalities.

**Table 1:** Laboratory investigations with neonatal hypocalcaemia

Investigations	Patient values	Normal values
Serum calcium	1.15	2.15–2.75 mmol/l
Ionised calcium	0.72	1.1–1.4 mmol/l
Serum phosphate	4.07	1.4–2.6 mmol/l
Serum magnesium	0.47	0.7–1.1 mmol/l
Albumin	37	25–40gm/L
PTH	<0.3	0.7–7pmol/l
Alkaline phosphatase	251	100–420U/l
Vitamin D	98	>50 nmol/l

**Fig. 3:**
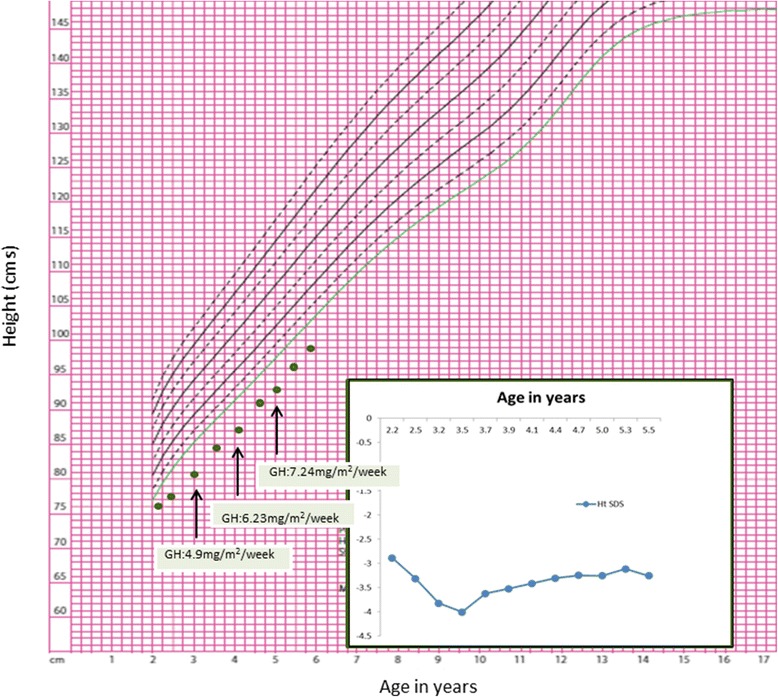
Figure 3 demonstrates the growth and the response of the patient to growth hormone (*plotted on the CDC growth chart*). Inset: Height SDS following growth hormone therapy

**Fig. 4:**
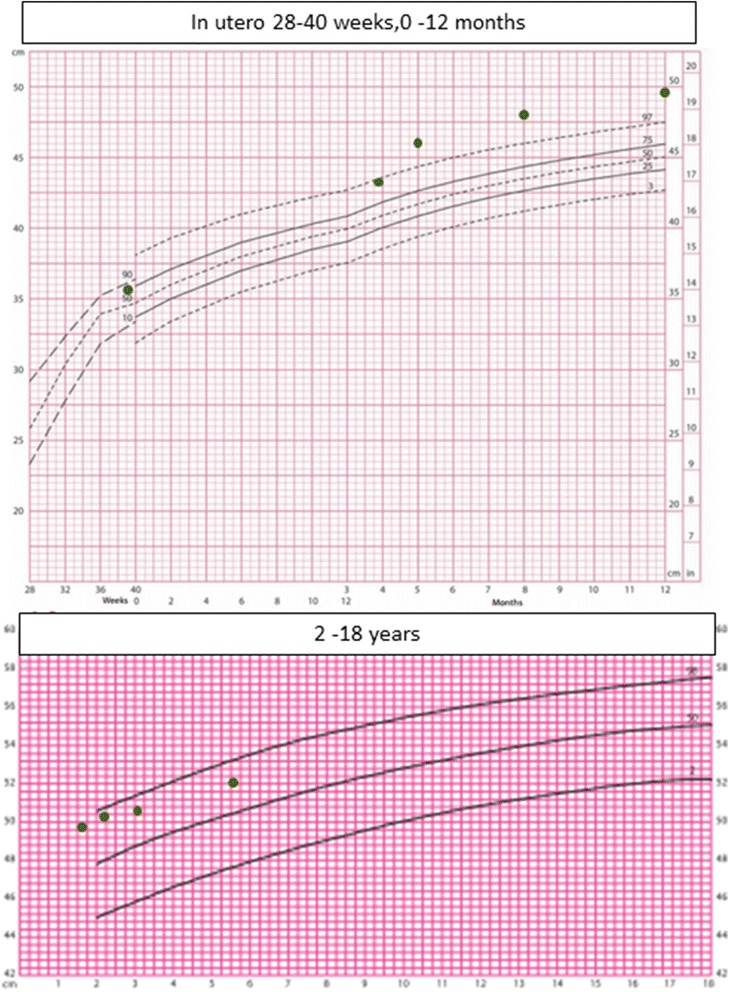
Figure 4 shows the head circumference of the patient

**Fig. 5:**
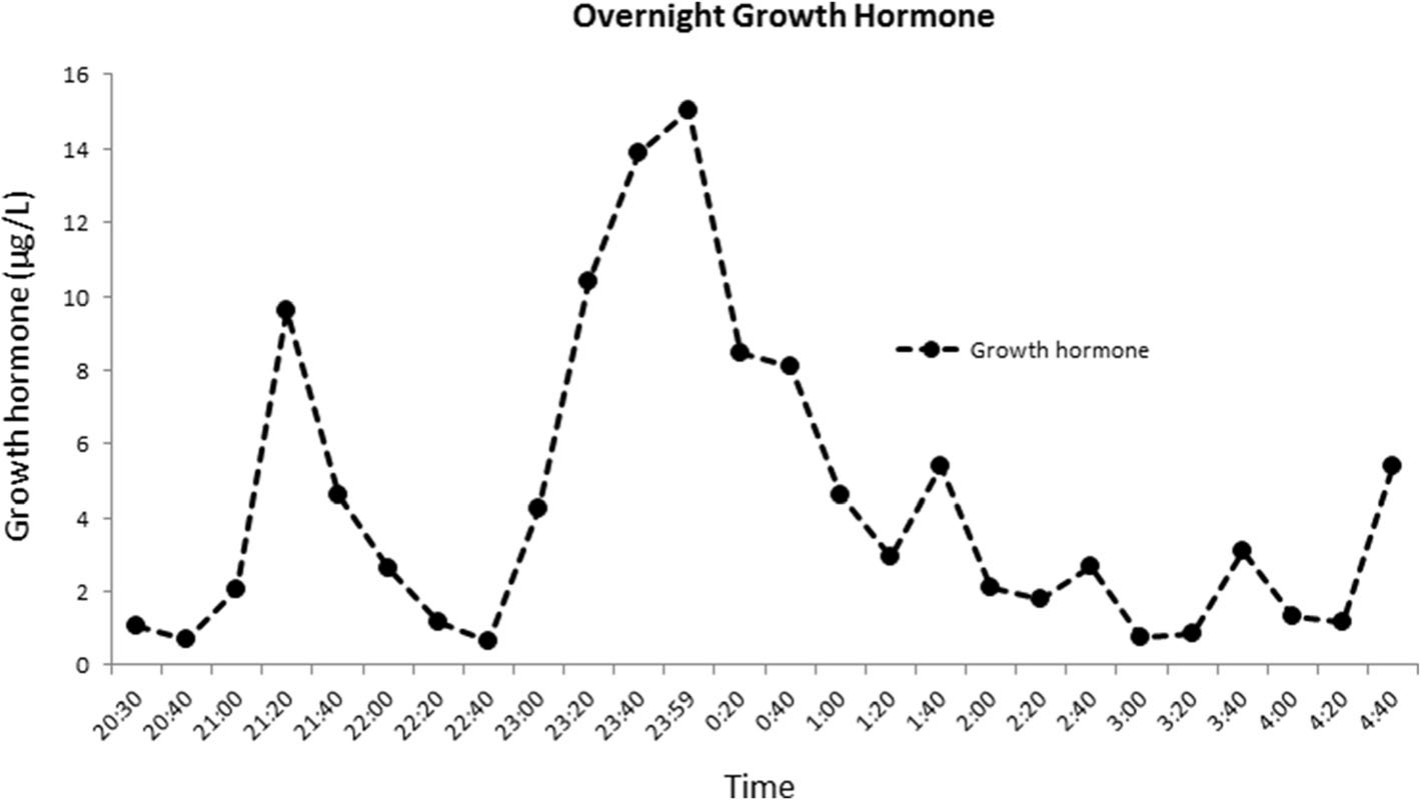
Figure 5 shows the overnight growth hormone (*GH*) test. Blood sample is collected every 20 min for GH and the physiological surge in GH is profiled. GH levels during waking hours are normally low. During sleep, there are usually several pulses of GH >20 mU/L (7.7 μg/L), usually associated with slow wave sleep. A peak GH response <10 mU/L (3.9 μg/L) suggests GH deficiency; a response of 10–20 mU/L (3.9–7.7 μg/L) may suggest partial GH deficiency; a response >20 mU/L (>7.7 μg/L) is regarded as normal.(1 mU/L × 0.385 = 1 μg/L)

Her neurodevelopment was normal. However, linear growth was poor (Fig. 1c) and dentition delayed; her first tooth erupted at 16 months. At 1 year of age, her length was 65.5cms (SDS −3.5) Figs. 3 and 4 demonstrate the height and head circumference respectively. Macrocrania was more pronounced during infancy and plateaued after 2 years of age although the head circumference continued to be disproportionate to the height. There was no family history of short stature, macrocephaly, skeletal and nail deformities or hypoparathyroidism. Parents were clinically unaffected with maternal height of 162.5cms and paternal height of 182.9cms, the predicted mid-parental target height was 166.2cms (SDS 0.4). Figure 5 demonstrates the overnight growth hormone (GH) study [6] performed at 2.6 years of age. It showed a normal GH reserve with a peak of 15 μg/L with serum insulin-like growth factor 1 (IGF-1) of 57 μg/L (SDS −1.9). GH was measured using a solid-phase, two-site chemiluminescent immunoassay on the Immulite 2000 XPi (Siemens Healthcare Diagnostics Inc, Deerfield, IL). IGF-1 was measured by chemiluminescent immunoassay on the automated analyser Diasorin Liaison (Diasorin Inc, Stillwater, MN). At 3 years and 2 months, her height was 80cms (SDS −3.86) (<3^rd^ percentile on WHO growth chart, <1^st^ percentile on CDC growth chart) with a height velocity of 5cms/year. GH treatment was commenced at 3 years and 3 months with a starting dose of 4.9 mg/m^2^/week (0.2 mg/kg/week) [7], under the Pharmaceutical Benefits Scheme Growth Hormone Program for ‘Short stature and slow growth’ [8]. This category utilises the following auxological parameters on the CDC growth charts; height <1^st^ percentile on the CDC growth chart and low height velocity (<3^rd^ percentile CDC growth chart). There were no side effects and good compliance with administration of injections was maintained.

**Fig. 6:**
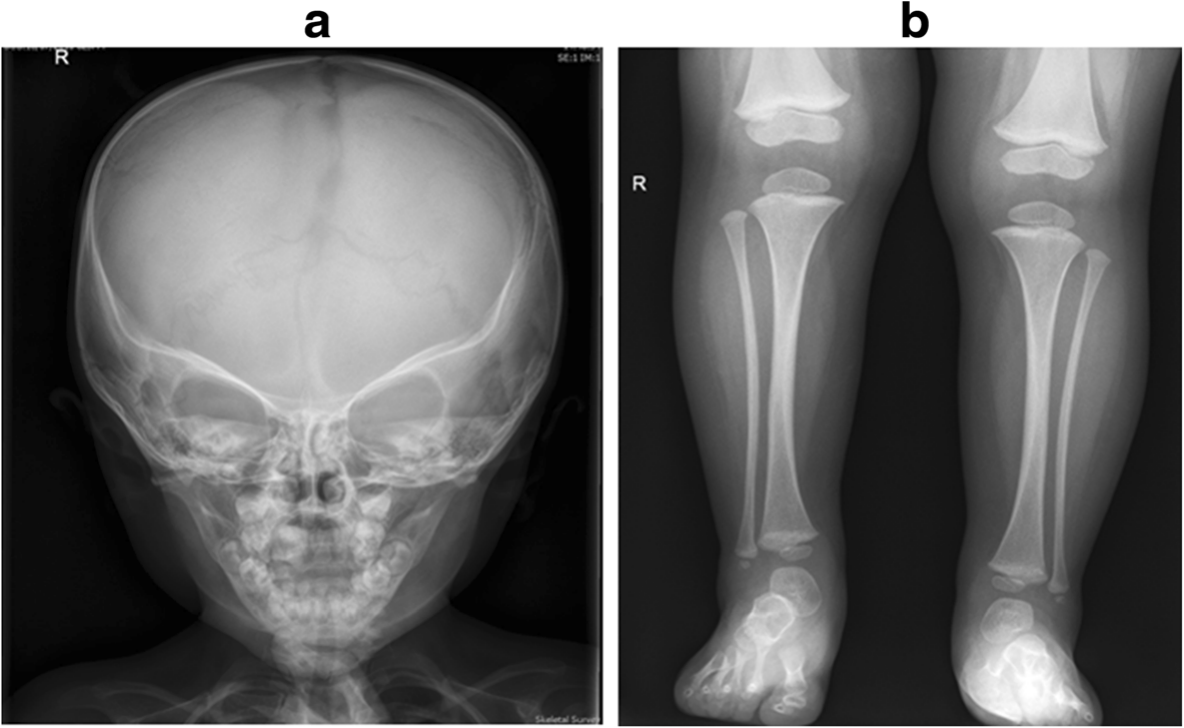
Figure 6**a** shows relatively poor ossification of skull vault, patent metopic suture, widely separated sagittal sutures on anteroposterior view of X-ray Skull. Figure 6**b** shows tubulated long bones with reduced medullary space and cortical thickening on anteroposterior view X-ray Tibia

**Fig. 7:**
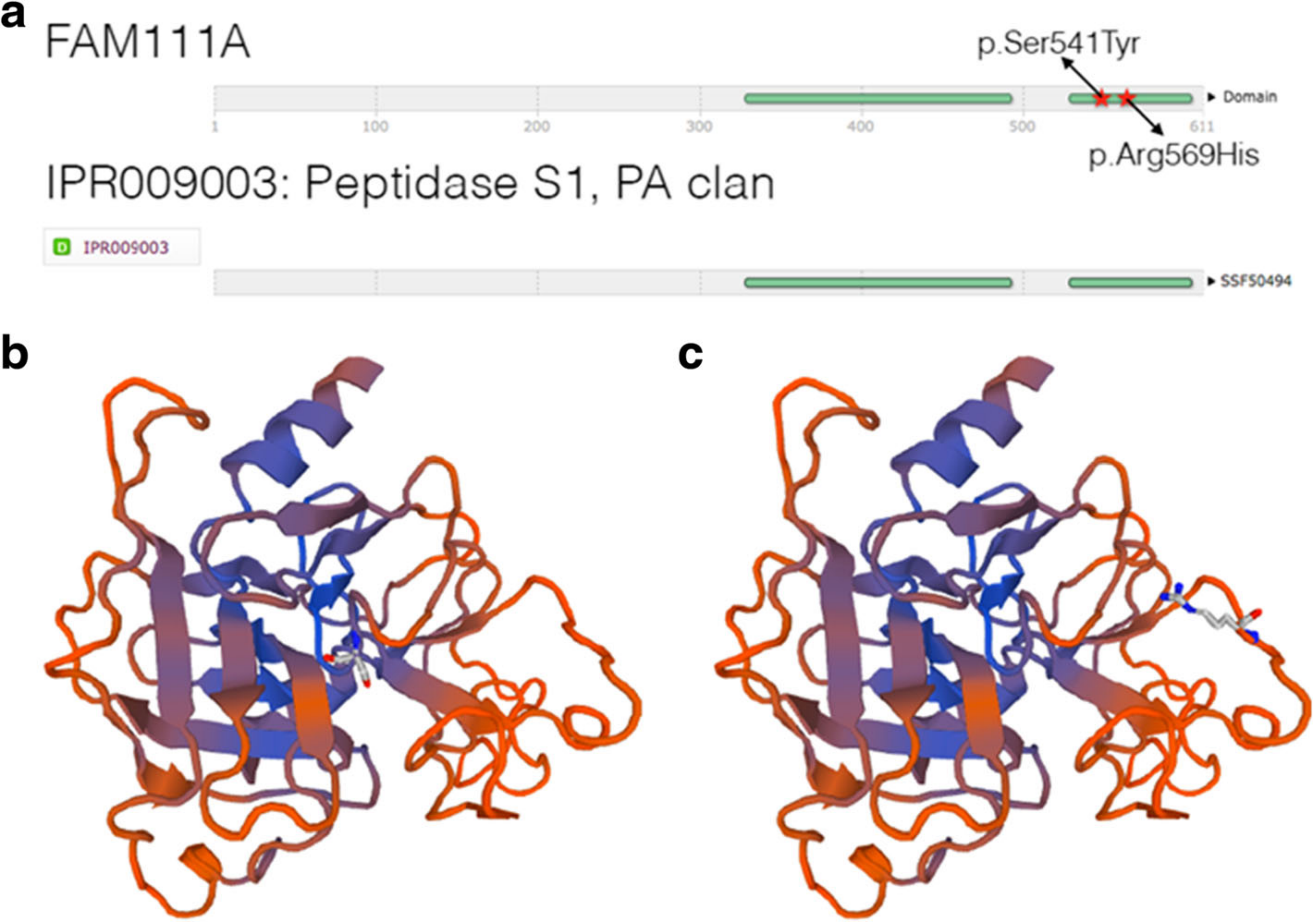
Figure 7**a** shows the FAM111A protein and Figures 7**b** and **c** show the 3D model. **a** The FAM111A protein is made up of 611 amino acid residues. Residues 329 to 492 and 529 to 603 are part of an InterPro domain named Peptidase S1, PA clan (*IPR009003*). Our novel variant p.Ser541Tyr lies in the same domain as a previously identified genetic variant, p.Arg569His, which has been associated with KCS Type 2. **b** and **c** SWISS-MODEL was used to generate a putative 3D model of FAM111A using the model template 4ic6.1, a protease that had the highest sequence identity (23.79%) to FAM111A. Only the peptidase domain of FAM111A could be modelled. Residue Ser541 is shown in 5B and Arg569 in Figure 7**c**
- *Follow-up*: Hypoparathyroidism was treated with oral calcium and calcitriol supplementation. Apart from early echogenic changes in the renal pelvis at 2 years of age, there has been no evidence of nephrocalcinosis or nephrolithiasis on follow-up at 4.6 years of age with normal renal function and urinary calcium excretion.

- *Investigations*: Initial investigations included a normal echocardiogram, renal ultrasound and MRI brain. There was also a persistent normocytic anaemia with haemoglobin of 95gm/L (NR: 110–145) with normal iron stores and haemoglobin electrophoresis. Karyotype and FISH for 22q11.2 deletion were normal. There was no pathogenic variant demonstrated on *CASR*, *PTH* and *GCMB* gene analysis. Chromosomal microarray revealed a small paternally inherited micro-duplication of chromosome 17p13.2 not known to cause any of the described phenotypic features. The proband’s father is asymptomatic, not dysmorphic and has normal calcium and PTH levels. A skeletal survey performed in the neonatal period was inconclusive; however, the repeat survey at 2 years of age was suggestive of KCS with overtubulated long bones, metacarpals and metatarsals with very little medullary space as shown in Fig. 6. The anterior fontanelle was widely patent with multiple Wormian bones. Further genetic testing revealed a novel *de novo* heterozygous mutation c.1622C > A (p. Ser541Tyr) in *FAM111A*. This genetic variant was predicted to be probably damaging by PolyPhen (score 0.99) [9], deleterious by SIFT (score 0.0) [10] and damaging by FATHMM (score −3.50) [11] suggesting that the mutation may be damaging. Furthermore, this variant is not present in the ExAC [12], 1000 Genomes Project [13], and NHLBI GO Exome Sequencing Project databases [14], supporting the putative dominant effect of this mutation. The variant lies in the peptidase domain of *FAM111A*, where a pathogenic variant for KCS type 2 was previously identified [4, 15] as shown in Fig. 7.

Figure 3 demonstrates the response to GH treatment. After the first year of GH treatment on 4.9 mg/m^2^/week (0.2 mg/kg/week), her height was 86.9cms (SDS −3.7), with a height velocity of 6.5cms/year. Serum IGF-1 levels increased from 52 μg/L (−1.9 SDS) to 154 μg/L (−0.2 SDS) during this period. The GH dose was increased to 6.23 mg/m^2^/week with a corresponding increase in IGF-1 to 193 μg/L (0.45 SDS) with further increment of doses (7.24 mg/m^2^/week or 0.33 mg/kg/week) in the next eight months. Her height velocity was 7.2cms/year at the end of second year of treatment with serum IGF-1 of 128 μg/L (−0.63 SDS). At 5.11 years, her weight is 14kgs and height is 98.9cms (SDS −3.18).

## Conclusions

The case highlights the clinical characteristics in a child with KCS Type 2 due to a novel *FAM111A* mutation. Hypoparathyroidism was diagnosed in the neonatal period and near normocalcemia was achieved with calcium and calcitriol supplements. Investigations ruled out the more commonly encountered DiGeorge syndrome and mutations in the less common *PTH* and *CASR* genes affecting PTH secretion, and *GCMB* gene related to parathyroid embryogenesis. Our proband did not have genetic confirmation of her condition until 4 years of age. We considered the diagnosis of KCS Type 2 based on her clinical and radiological features. The neonatal skeletal survey was inconclusive; however, a survey repeated at 2 years of age raised the possibility of KCS with the evidence of medullary stenosis. She had additional features including macrocrania with widely separated sutures, dysmorphic features, normal intellect and developmental milestones. The absence of intellectual impairment and microcephaly excluded KCS Type 1 and Sanjad Sakati syndrome. The genetic cause of KCS Type 2 was first reported by Unger et al., and involves the “Family with sequence similarity 111, member A00 (*FAM111A*) gene (NM_001142519.1)” as reported in five patients with KCS Type 2 and five patients with severe osteocraniostenosis (OCS) [4]. KCS type 2 and OCS were allelic disorders of different severity with each having a close genotype-phenotype correlation. The p.Arg569His (R569H) mutation was consistently associated with the KCS phenotype while another mutation, p.Ser342del, was associated with the lethal phenotype of OCS. Japanese researchers also identified the same mutation independently in another four patients and they concluded that R569H is a hot spot mutation for KCS Type 2 [15]. Our proband has a novel *FAM111A* mutation (p. Ser541Tyr), which lies in the same peptidase domain as the hot spot mutation. Within this domain, the S541 residue, along with H385, and D439 forms the putative catalytic triad of *FAM111A* [4]. As a consequence, the mutation in our proband may impair the catalytic activity of *FAM111A*. The identified cases from the two above-mentioned series were *de novo* mutations [4, 15]. However, the earliest description of KCS described a mother-to-son transmission [16] and this has been further elaborated in a second case of mother-to-daughter transmission of the same mutation with both mother and child affected [17] consistent with an autosomal dominant inheritance.

*FAM111A* has very low overall sequence identity to known proteins; however, the second half of the protein has homology to a family of peptidases (IPR009003). We generated a 3D protein model of *FAM111A* (Fig. 5b and c) using a known protease with the highest sequence similarity to *FAM111A* (SWISS-MODEL template ID 4ic6.1) [18]. The S541 residue appears to lie closer to the core of the protein, consistent with its predicted role as part of the catalytic triad (Fig. 5b) whereas the R569 residue appears to be on the outer surface of the protein (Fig. 5c), as previously reported by Unger and colleagues [4]. Furthermore, our functional predictions of Ser541Tyr suggest that the variant may have very damaging effects, in contrast to the tolerated and benign predictions made on Arg569His [9]. Taken together, the pathogenicity of the novel *FAM111A* mutation in our proband may be different to the previously identified hot spot mutation.

*FAM111A* is constitutively expressed in bone and parathyroid gland and may have a role affecting intracellular pathways regulating normal bone development, height gain, and parathyroid gland development and/or regulation [4, 15]; although the precise mechanisms are unknown. *FAM111A* demonstrates nuclear localization and interacts with the C-terminal of SV40 large T antigen to promote viral replication in a transfected human osteosarcoma cell line (U2OS) [19]. Studies evaluating chromatin during DNA replication have identified interaction between *FAM111A* and proliferating cell nuclear antigen, a key part of the complex involved in stabilization of DNA polymerases during replication [20, 21]. Depletion of *FAM111A* in U2OS cells resulted in delayed entry into S-phase and DNA synthesis [19]. The interaction of GH signalling with *FAM111A* function either directly or indirectly in utero and postnatally is not yet elucidated. This will be an important area of future investigation.

Patients with *FAM111A* mutations have severe short stature (−3 to −7 SDS) [4]. KCS has been identified as a cause for short stature with dysmorphic features in the absence of hypoparathyroidism [22]. Our proband did not exhibit prenatal growth restriction and growth faltered in the first year of life. This is similar to the other reported cases and hence it suggests that *FAM111A* is predominantly involved with postnatal growth [9]. The slow growth in our proband was striking in the absence of dietary issues, coeliac disease or any other chronic illness. The anaemia, although persistent, was mild and hence unlikely to contribute to the severity of growth restriction observed in our patient. She was eligible for GH therapy under the ‘Short stature and slow growth’ category in Australia [8]. We initiated GH therapy at 3 years and 3 months of age with the intention of improving final height despite normal constitutive GH levels. There has been one other report of the use of GH (dose unspecified) in a female patient with KCS Type 2 in whom GH was commenced at 3.6 years for short stature [22, 23]. An initial response in growth velocity was not sustained in the latter patient and therapy was changed over to IGF-1 therapy, which was subsequently discontinued due to severe headaches, poor compliance and poor growth response. Reintroduction of GH treatment improved her growth velocity. The height SDS improved from −5.99 at baseline to −3.38 at 12 years and 1 month [22]. In our patient, there was reportedly good compliance to treatment throughout therapy and the height SDS improved from −3.86 to −3.18. During her first year of treatment, the height velocity of 6.5cms/year, was below −1 SDS on the Bakker’s curve [24]. The GH dose of 4.5 mg/m^2^/week (0.17 mg/kg/week) is the standard dose used for commencement of GH therapy in Australia; this is lower than that used in other countries (0.3 mg/kg/week). There was an improvement in height velocity in the second year of treatment as the GH dose increased, although the response remained suboptimal even after therapy increased to 0.3 mg/kg/week as demonstrated in Fig. 3. It is not known if doses above 0.3 mg/kg/week would have been effective at improving growth velocity, as she was not eligible for higher GH doses at the time of her treatment under the Australian Growth Hormone Programme funded by the Pharmaceutical Benefits Scheme.

Many children with genetic and syndromic conditions are eligible for GH therapy under the category of ‘Short stature and slow growth’ in Australia. This group forms the largest category of patients treated with GH during childhood. In an observational study (1990–2013) of the Australian Growth Hormone Programme, rates of non-compliance approached 50% and early cessation of GH treatment was noted [24]. The expense of treatment [25] and the unknown long-term effects increase the complexity of management [26] of patients with short stature. Normal variation in height extends beyond the growth hormone-insulin-like growth factor 1 (GH-IGF-1) axis [27]. Short stature in KCS Type 2 is not due to perturbation of the GH-IGF-1 axis and is likely to be associated with a skeletal defect that limits growth. Recent advances in sequencing have enabled more rapid identification of the genetic etiology in children with short stature and support newer diagnostic and therapeutic paradigms centred on understanding the genetics of the growth plate and skeletal development [27]. These may enable better targeting of GH treatment in children with short stature. Case reports such as these provide important information for families and raise the issue of the prioritization and costs of genetic tests prior to GH therapy for short stature.

Although limited to a single case, this report aims to provide clinicians with information regarding growth response of KCS Type 2 with GH therapy. We propose that clinical case reports such as ours and collaboration between clinicians to establish and support international registries monitoring and reporting the outcome of GH therapy in rare genetic conditions such as KCS would be helpful in moving towards personalised medicine and guiding clinical decisions about the use and doses of GH in short stature.

## Abbreviations

GH: Growth hormone
IGF-1: Insulin-like growth factor 1
KCS: Kenny-Caffey syndrome
OCS: Osteocraniostenosis
PTH: Parathyroid hormone
SDS: Standard deviation score

## References

[1] Cho WI, Yu HW, Chung HR (2015). Clinical and laboratory characteristics of neonatal hypocalcemia. Ann pediatr endocrinol metab.

[2] Park SY, Eom YS, Choi B (2013). Genetic and clinical characteristics of Korean patients with isolated hypoparathyroidism: from the Korean hypopara registry study. J Korean med sci.

[3] Kim JH, Shin YL, Yang S (2015). Diverse genetic aetiologies and clinical outcomes of paediatric hypoparathyroidism. Clin endocrinol (Oxf).

[4] Unger S, Gorna MW, Le Bechec A (2013). FAM111A mutations result in hypoparathyroidism and impaired skeletal development. Am j hum genet.

[5] Parvari R, Hershkovitz E, Grossman N (2002). Mutation of TBCE causes hypoparathyroidism-retardation-dysmorphism and autosomal recessive Kenny-Caffey syndrome. Nat genet.

[6] Honda Y, Takahashi K, Takahashi S, et al. Growth hormone secretion during nocturnal sleep in normal subjects. The Journal of clinical endocrinology and metabolism (1969);29: 20–29 10.1210/jcem-29-1-20 4302913

[7] Hughes IP, Harris M, Cotterill A (2014). Comparison of weight- vs body surface area-based growth hormone dosing for children: implications for response. Clin endocrinol (Oxf).

[8] Department of Health:Guidelines for the Pharmaceutical Benefit Scheme Growth HormoneProgramme. Available from http://www.health.gov.au/hgh. Accessed 11 Nov 2014.

[9] Adzhubei IA, Schmidt S, Peshkin L (2010). A method and server for predicting damaging missense mutations. Nat methods.

[10] Kumar P, Henikoff S, Ng PC (2009). Predicting the effects of coding non-synonymous variants on protein function using the SIFT algorithm. Nat protoc.

[11] Shihab HA, Gough J, Cooper DN (2013). Predicting the functional, molecular, and phenotypic consequences of amino acid substitutions using hidden Markov models. Hum mutat.

[12] Lek M, Karczewski KJ, Minikel EV (2016). Analysis of protein-coding genetic variation in 60,706 humans. Nature.

[13] Auton A, Brooks LD, Durbin RM (2015). A global reference for human genetic variation. Nature.

[14] Tennessen JA, Bigham AW, O’Connor TD (2012). Evolution and functional impact of rare coding variation from deep sequencing of human exomes. Science (New York, NY).

[15] Isojima T, Doi K, Mitsui J (2014). A recurrent de novo FAM111A mutation causes Kenny-Caffey syndrome type 2. J bone miner res off j am soc bone miner res.

[16] Kenny FM, Linarelli L (1966). Dwarfism and cortical thickening of tubular bones. Transient hypocalcemia in a mother and son. Am j dis child.

[17] Nikkel SM, Ahmed A, Smith A, Marcadier J, Bulman DE, Boycott KM (2014). Mother-to-daughter transmission of Kenny-Caffey syndrome associated with the recurrent, dominant FAM111A mutation p.Arg569His. Clin genet.

[18] Biasini M, Bienert S, Waterhouse A (2014). SWISS-MODEL: modelling protein tertiary and quaternary structure using evolutionary information. Nucleic acids res.

[19] Fine DA, Rozenblatt-Rosen O, Padi M (2012). Identification of FAM111A as an SV40 host range restriction and adenovirus helper factor. Plos pathog.

[20] Alabert C, Bukowski-Wills JC, Lee SB (2014). Nascent chromatin capture proteomics determines chromatin dynamics during DNA replication and identifies unknown fork components. Nat cell biol.

[21] Bowman GD, O’Donnell M, Kuriyan J (2004). Structural analysis of a eukaryotic sliding DNA clamp-clamp loader complex. Nature.

[22] Guo MH, Shen Y, Walvoord EC (2014). Whole exome sequencing to identify genetic causes of short stature. Horm res paediatr.

[23] Bauters M, Guo M, Dauber A, Walvoord E (2014). Kenny-Caffey syndrome: an expanded phenotype and response to growth hormone therapy.

[24] Hughes IP, Choong C, Rath S (2016). Early cessation and non-response are important and possibly related problems in growth hormone therapy: an OZGROW analysis. Growth hormon IGF res off j growth hormon res soc int IGF res soc.

[25] Allen DB, Cuttler L (2013). Clinical practice. Short stature in childhood—challenges and choices. N engl j med.

[26] Collett-Solberg PF (2011). Update in growth hormone therapy of children. J clin endocrinol metab.

[27] Baron J, Savendahl L, De Luca F (2015). Short and tall stature: a new paradigm emerges. Nat rev endocrinol.

